# Kaposi's Sarcoma-Associated Herpesvirus (KSHV) vIL-6 Promotes Cell Proliferation and Migration by Upregulating DNMT1 via STAT3 Activation

**DOI:** 10.1371/journal.pone.0093478

**Published:** 2014-03-27

**Authors:** Jing Wu, Yuqiao Xu, Dongping Mo, Peijun Huang, Ruihong Sun, Lei Huang, Shiyang Pan, Jian Xu

**Affiliations:** 1 Department of Respiratory Medicine, Nanjing Chest Hospital, Nanjing, China; 2 Department of Laboratory Medicine, The First Affiliated Hospital of Nanjing Medical University, Nanjing, China; 3 National Key Clinical Department of Laboratory Medicine, Nanjing, China; University of Southern California Keck School of Medicine, United States of America

## Abstract

Kaposi's sarcoma-associated herpesvirus (KSHV) is etiologically associated with Kaposi's sarcoma (KS), the most common AIDS-related malignancy. KSHV vIL-6 promotes KS development, but the exact mechanisms remain unclear. Here, we reported that KSHV vIL-6 enhanced the expression of DNA methyltransferase 1 (DNMT1) in endothelial cells,increased the global genomic DNA methylation, and promoted cell proliferation and migration. And this effect could be blocked by the DNA methyltransferase inhibitor, 5-azadeoxycytidine. We also showed that vIL-6 induced up-regulation of DNMT1 was dependent on STAT3 activation. Therefore, the present study suggests that vIL-6 plays a role in KS tumorigenesis partly by activating DNMT1 and inducing aberrant DNA methylation, and it might be a potential target for KS therapy.

## Introduction

Kaposi's sarcoma-associated herpesvirus (KSHV), also known as human herpesvirus 8 (HHV-8), is causally linked to Kaposi's sarcoma (KS) [1], primary effusion lymphoma (PEL) [2],and multicentric Castleman's disease (MCD) [3]. KS is an endothelial cell-derived tumor, the most common neoplasm in untreated human immunodeficiency virus (HIV)-infected individuals [4]. Acquired immunodeficiency syndrome-associated KS (AIDS-KS) has been one of the most frequent cancers affecting men and children in many subequatorial African countries, where it is associated with significant morbidity and mortality. Furthermore, although the incidence of AIDS-KS in the developed countries has declined since the widespread use of highly active antiretroviral treatment (HAART), it can not be cured yet, and nearly 50% of patients never achieve total remission [5].

The mechanisms of KS pathogenesis by KSHV have not been fully elucidated. Most of the studies focused on KSHV genes with oncogenic properties. KSHV encodes more than 85 protein-coding genes, and at least two dozen microRNAs [6]. Among them, ORF K2 encodes a viral form of interleukin-6 (vIL-6), which can promote cellular proliferation and survival by activating STAT3 and AKT signaling pathways [7]–[9]. In addition, it can also induce the secretion of cellular IL-6 and VEGF to enhance tumorigenesis, angiogenesis and hematopoiesis [10]. vIL-6 is a homologue of cellular IL-6. vIL-6 shares 25% amino-acid identity with human cellular IL-6 [11]. Multiple evidences demonstrated that cellular IL-6 could promote tumorigenesis by activating DNMT1, inducing aberrant DNA methylation and affecting gene expression [12]–[16]. Given the structural and functional similarity between vIL-6 and cellular IL-6, we hypothesize that vIL-6 could activate DNMT1, affect genomic methylation, and therefore contribute to the tumorigenesis of KS.

## Materials and Methods

### Cells, plasmids and Reagents

The human endothelial cell line EA.hy926 is derived by fusing human umbilical vein endothelial cells with the permanent human cell line A549 [17], and it retains expression of several endothelial cell markers and properties [18]. It was purchased from Shanghai Institute of Cell Biology, Chinese Academy of Sciences, and cultured in a humidified 5% CO2 atmosphere at 37°C in DMEM containing 10% fetal bovine serum (FBS), 100 U/ml penicillin and 100 mg/ml streptomycin. In addition, we generated lines of EA.hy926 cells that express vIL-6 by transducing a lentivirus containing KSHV K2 gene. Transduction of empty lentiviral vector served as a negative control. Stable lines were selected with hygromycin. The expression of vIL-6 protein in EA.hy926 cell clones was detected by Western blot. Finally, ten stable transfectants were obtained; representative clones E4, E8, and the negative control E0 were used in this study. The dominant negative construct of STAT3 (pMSCV-STAT3-DN), and corresponding control vector pMSCV were kindly provided by Prof. Daniel C. Link (Washington University School of Medicine, USA) [19]. The STAT3 inhibitor, S3I-201, was from Santa Cruz Biotechnology, and the AKT inhibitor IV was from Calbiochem. DNMT1 inhibitor 5-azadeoxycytidine (5-aza-CdR) was from Sigma. Lipofectamine 2000 reagent was from Invitrogen.

### Western blotting

Western blotting was performed as previously described [20]. The primary antibodies and their final dilutions were as follows: vIL-6 (1∶500; Abbiotec), DNMT1 (1∶1,000; Cell Signaling), STAT3 (1∶1,000; Cell Signaling), phospho-STAT3 (1∶1,000; Cell Signaling), AKT (1∶1,000; Cell Signaling), phospho-AKT (1∶1,000; Cell Signaling), actin (1∶1,000; Santa Cruz Biotechnology), lamin A/C (1∶1,000; Cell Signaling).

### Cell proliferation assay

Cells were treated with or without 1 μmol/L 5-aza-CdR as indicated, seeded into 96-well plates and incubated. Proliferation was assessed by 3-(4,5-dimethylthiazol-2-yl)-2,5-diphenyltetrazolium bromide (MTT) assay according to standard methods.

### Matrigel invasion assay

The Matrigel invasion assay was performed according to the manufacturer's instructions (BD Biosciences). Briefly, 1×10^4^ cells in 0.5 mL of medium containing 1% FBS were added to the transwell insert, which was seated in 750 μL of complete medium (10% FBS). After a 24-h incubation, noninvading cells were mechanically removed. Cells that had migrated through the Matrigel were stained with the Diff-Quick staining kit (Dade Behring). Cells were counted in five representative microscopic fields (×200 magnification) and photographed.

### The measurement of DNMT1 enzyme activity

Nuclear proteins were measured by BCA method (Thermo scientific), and then DNMT1 enzyme activity was assessed using EpiQuik DNMT Activity/Inhibition Assay Ultra Kit (Epigentek) according to the manufacturer's protocol. All samples were run in triplicates and the average was used for analysis.

### Global DNA methylation analysis

Genomic DNA purified from cells was used for quantification of methylated DNA using the MethylFlash Methylated DNA Quantification Kit (Epigentek) according to the manufacturer's instructions. All samples were run in triplicates and the average was used for analysis.

### Statistical analysis

Data are presented as mean±SD. Statistical significance was determined with a 2-tailed Student *t* test.

## Results

### vIL-6 promotes EA.hy926 cell proliferation and migration

**Figure 1:**
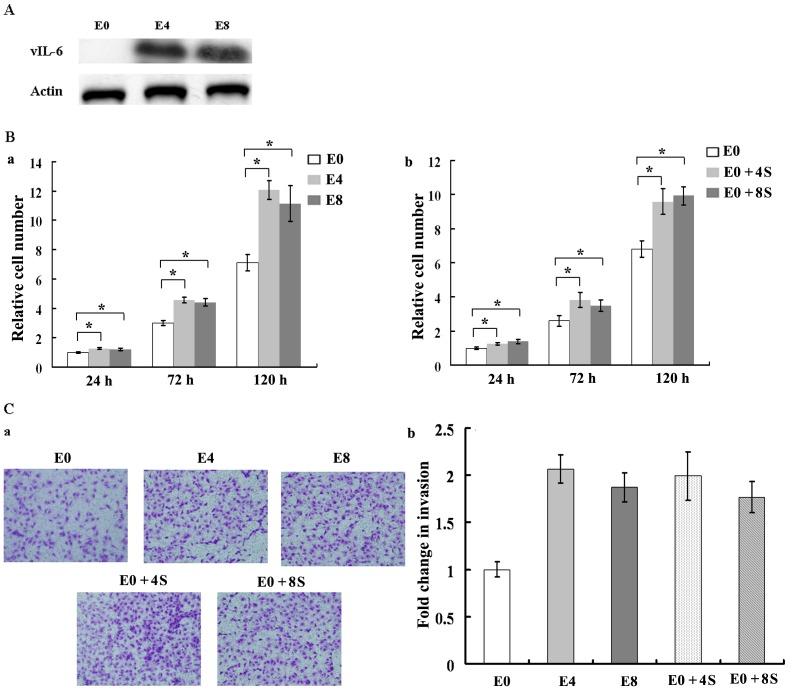
vIL-6 promotes EA.hy926 cell proliferation and migration. A, Western blot analysis of vIL-6 proteins in lentivirus-transduced EA.hy926 cells. B, vIL-6 promotes cell proliferation. Cell viability was assessed by MTT assay. Results were normalized to that of control and are means of 5 experiments ± SD. a, vIL-6 promoted the proliferation of EA.hy926 cells by autocrine pathway. b, vIL-6 promoted the proliferation of EA.hy926 cells by paracrine pathway. C, vIL-6 promotes cell migration. a, Matrigel invasion assay. Photomicrographs of cells that have passed through Matrigel. Original magnification, ×200. b, quantification of invasion. The number of cells that migrated through pores onto the lower side of the filter was counted. We compared the number of cells that migrated with the number of untreated E0 cells that migrated. The Y axis represents fold change of invasiveness (see Materials and Methods for details). E0, EA.hy926 cells transduced with an empty lentiviral vector; E4 and E8, two cell clones of EA.hy926 cells transduced with a lentivirus containing KSHV K2 gene. E0+4S, E0 cells cultured in E4 cell culture supernatant; E0+8S, E0 cells cultured in E8 cell culture supernatant. *, *P*<0.05.

To investigate the effect of vIL-6 on EA.hy926 cells, we transduced the cells with a lentivirus encoding vIL-6. Western blot showed that vIL-6 was highly expressed in both E4 and E8 clones (Fig. 1A). We examined the effect of vIL-6 on the proliferation of EA.hy926 cells using MTT assay. vIL-6-expressing cells E4 and E8 had higher proliferation rates compared to E0 cells. Then we collected E4 and E8 cell culture supernatant and add it into E0 culture medium. E0 cells cultured in E4 or E8 culture supernatant also had higher proliferation rates than untreated E0 cells (Fig. 1B). To examine the effect of vIL-6 on cell migration, we performed Matrigel invasion assay. Striking differences were observed. Untreated E0 cells migrated through the Matrigel chamber in relatively low numbers, whereas E4, E8 and E0 cells cultured in E4 or E8 culture supernatant totally exhibited a marked increase in invasion in this assay at 24 hours (Fig. 1C). Taken together, these data indicate that vIL-6 promotes EA.hy926 cell proliferation, and migration by both autocrine and paracrine pathways.

### vIL-6 enhances DNMT1 expression and activity

**Figure 2:**
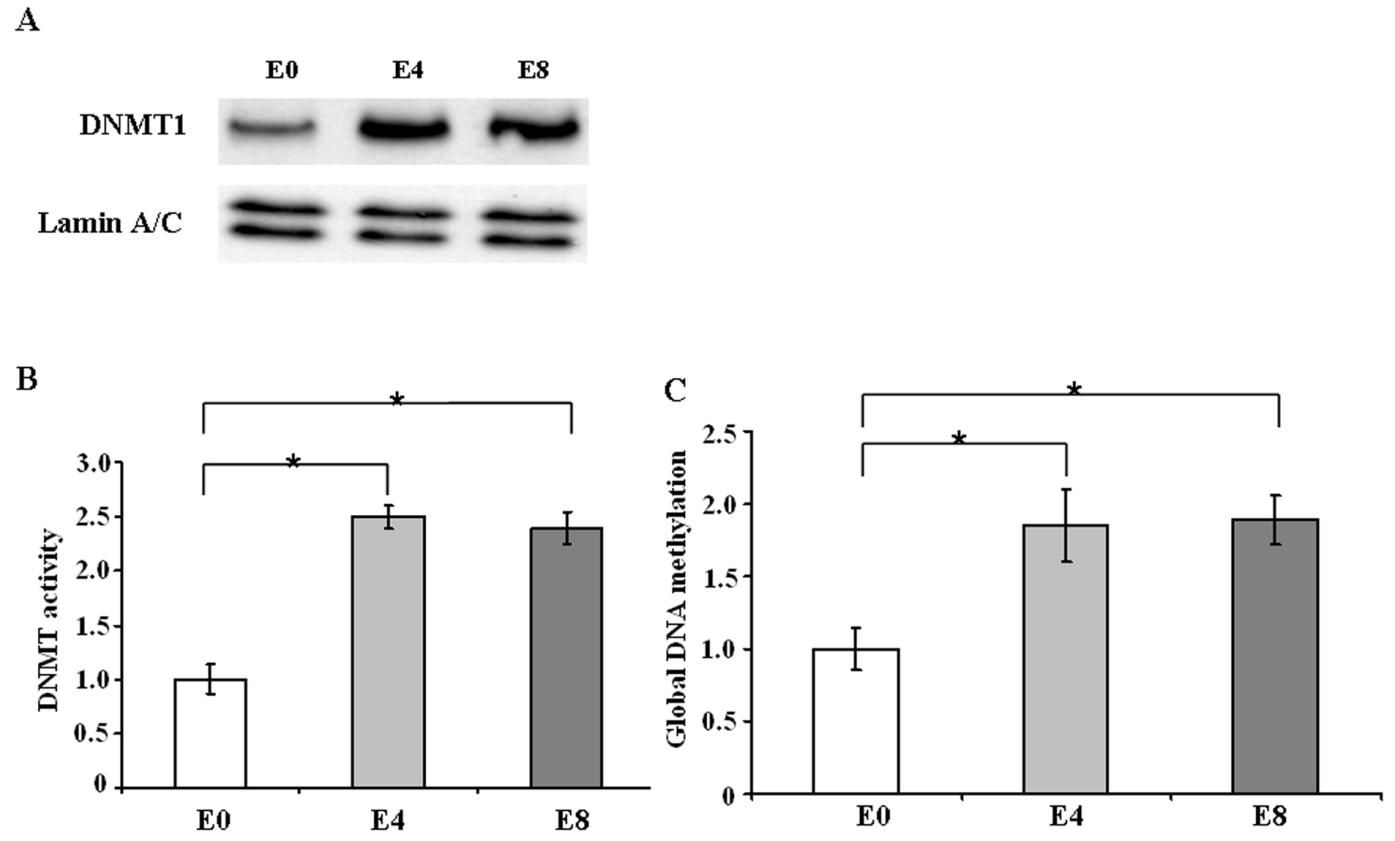
vIL-6 enhances DNMT1 expression and activity. A, Western blot analysis of DNMT1 proteins. B, quantification of enzyme activity of DNMT1. C, quantification of global DNA methylation. The results represent the mean ± SD of 3 independent experiments. *, *P*<0.05.

IL-6 is known to affect DNMT1 expression and activity. Since vIL-6 is the homologue of cellular IL-6, we tested the effect of vIL-6 on DNMT1. The expression level of DNMT1 was evaluated by Western blot. As shown in Fig. 2A, DNMT1 expression increased in vIL-6-expressing cells E4 and E8 than mock cells. We also aimed to determine the effect of vIL-6 on the enzymatic activity of DNMT1. As shown in Fig. 2B, vIL-6-expressing cells E4 and E8 had higher enzyme activity than the control. Thus, vIL-6 mediates an increase in DNMT1 expression level and its enzymatic activity. Next, global DNA methylation was assessed by an ELISA-like assay, using an antibody against 5-methylcytosine. Global DNA methylation was significantly increased in E4 and E8 cells as compared to the control (Fig. 2C), suggesting that vIL-6 expression leaded to an increase in global DNA methylation.

### 5-aza-CdR inhibits cell proliferation and migration

**Figure 3:**
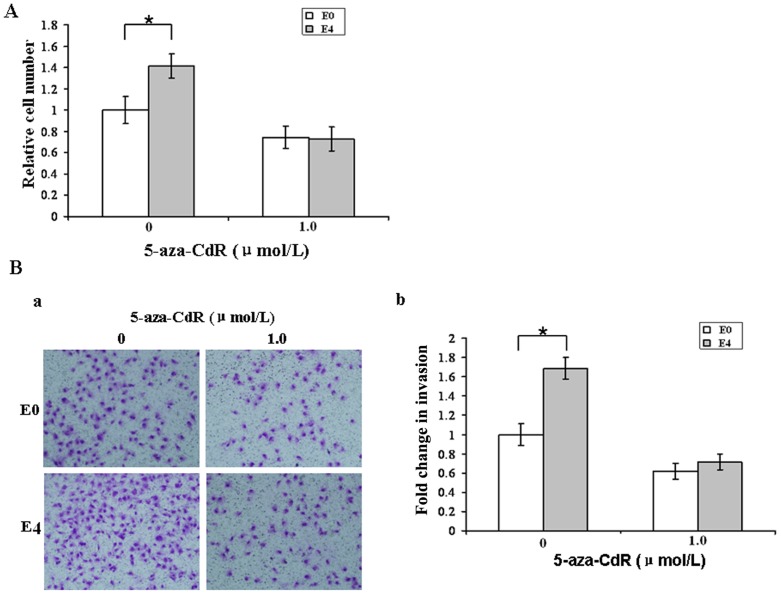
5-aza-CdR inhibits cell proliferation and migration. A, Cell viability was determined after 72 h incubation by MTT assay. Results were normalized to that of untreated control and are means of 5 experiments ± SD. B, Matrigel invasion assay. a, photomicrographs of cells that have passed through Matrigel. Original magnification, ×200. b, quantification of invasion. The number of cells that migrated through pores onto the lower side of the filter was counted. We compared the number of cells that migrated with the number of untreated E0 cells that migrated. The Y axis represents fold change of invasiveness (see Materials and Methods for details).*, *P*<0.05.

We sought to determine if the high proliferation and migration potential of vIL-6-expressing cells is due to increased expression and activity of DNMT1. As shown in Fig. 3A, the proliferation rate of vIL-6-expressing cells E4 was much higher than that of control E0 cells. However, after 72 hours of treatment, 5-aza-CdR lowered cell density of each group to a similar level. Second, we assessed the effect of 5-aza-CdR on Matrigel invasion. Without treatment of 5-aza-CdR, invading E4 cells were much more than invading E0 cells. After treatment, invading E4 cells decreased more dramatically, and no significant difference in invading cell number was observed between both cell groups (Fig. 3B). Together, these results indicate that vIL-6 promoted cell proliferation and migration by increasing the expression and activity of DNMT1.

### vIL-6 upregulates DNMT1 expression via STAT3 activation

**Figure 4:**
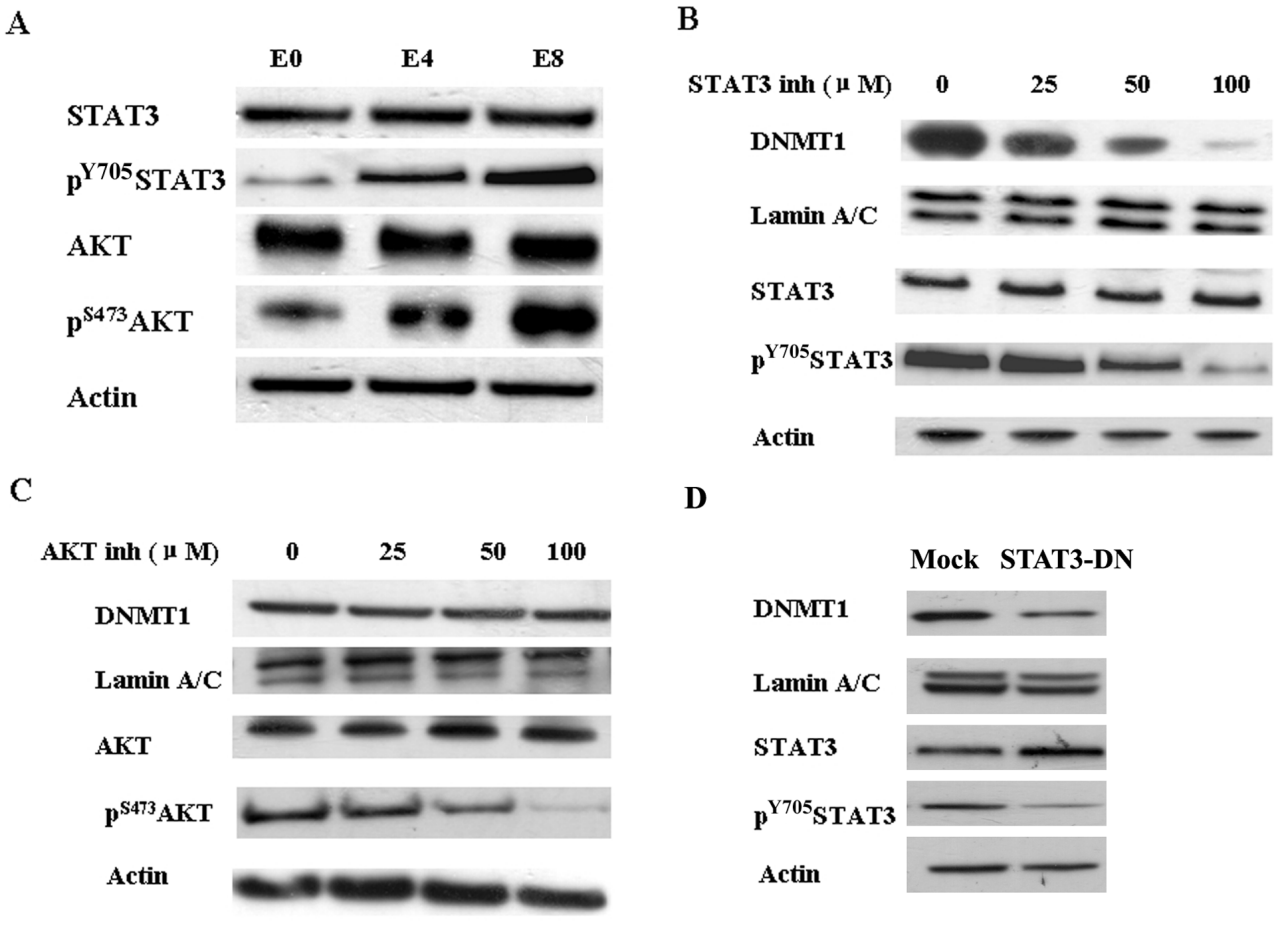
vIL-6 activates DNMT1 via STAT3. A, vIL-6 activated both STAT3 and AKT pathways. B, pharmacologic inhibition of STAT3 inhibits phosphorylation of STAT3 and DNMT1 expression in a dose dependent fashion. C, pharmacologic inhibition of AKT inhibits phosphorylation of AKT in a dose dependent fashion but does not influence expression of DNMT1. D, DNMT1 expression decreases after transfection of the dominant negative construct of STAT3 (pMSCV-STAT3-DN).

It was reported that vIL-6 could activate both STAT3 and AKT pathway. Thus, we sought to determine whether the activation of STAT3 and/or AKT was attributable to increased DNMT1 expression. As expected, vIL-6 enhanced the expression of both phosphorylated STAT3 and phosphorylated AKT in E4 and E8 cells (Fig. 4A). Inhibition of STAT3 activity by the STAT3 inhibitor resulted in reduced both phosphorylated STAT3 and DNMT1 expression in vIL-6-expressing cells E4, and the effects were dose dependent (Fig. 4B). However, although the AKT inhibitor decreased phosphorylated AKT expression with dose dependent manner, it did not affect DNMT1 expression (Fig. 4C). Furthermore, we transfected the dominant negative construct of STAT3 (pMSCV-STAT3-DN) and the control vector pMSCV into E4 cells respectively. DNMT1 expression decreased dramatically in STAT3-DN transfected cells (Fig. 4D). It indicates that vIL-6 increases DNMT1 expression via STAT3 activation.

## Discussion

DNA methylation, catalyzed by methyltransferases(DNMTs), has critical roles in the control of gene expression and cellular processes [21]. Aberrant expression of DNMTs and disruption of DNA methylation patterns are closely associated with many forms of cancer [22]. Cellular IL-6, a bridge molecule between chronic inflammation and carcinogenesis, could promote colonic tumorigenesis through DNMT1-mediated tumor suppressor gene hypermethylation [12]. Similar evidences were found in erythroleukemia [13], multiple myeloma [14], cholangiocarcinoma [15], as well as oral cancer [16]. KSHV contains more than 80 open reading frames (ORFs), some of which are pirated from the host genome in order to control cell signaling, proliferation and immune regulation [23]. In this study, we found that vIL-6, a homolog of cellular IL-6, could also promote oncogenesis by increasing DNMT1 expression.

Virus infection has been linked to increased expression of DNMTs in cells infected with hepatitis B virus [24], [25], Epstein–Barr virus [26] and human papillomavirus [27]. We reported here that KSHV vIL-6 could enhance the expression of DNMT1,and increase the global genomic DNA methylation. DNMT1 is responsible for both *de novo* and maintenance methylation [28]. Overexpression of DNMT1 in nontransformed cells leads to cellular transformation [29], whereas knockout of this gene protects mice from colorectal cancer [30]. Latency-associated nuclear antigen (LANA), another oncogenic gene of KSHV, could induce aberrant DNA methylation in a different way by recruiting *de novo* methyltransferase DNMT3a to the chromatin and targeting repression of approximately 80 cellular genes, some of which are known targets of epigenetic inactivation in various cancers [31]. The existing evidence shows that KSHV reprograms cellular gene expression by a serial of complex and deliberate epigenetic mechanisms. Besides DNA methylation, histone modifications, chromatin remodelling and microRNA processing are all involved [32], [33]. The effect of vIL-6 on DNMT1 described here,enriched our knowledge about the role of KSHV in epigenetic modification in cancer.

Several signaling pathways lead to overexpression of DNMT1 through transcriptional and posttranscriptional control mechanisms [34], [35]. Studies have reported that DNMT1 gene expression was controlled via transcription factor Fli-1 in erythroleukemia cells [13], that DNMT1 expression was increased through the JAK-STAT pathway in malignant T lymphocytes [36], and that AKT activity inhibited DNMT1 degradation in multiple cell lines [37]. Our results indicate that in vIL-6 over-expressed endothelial cells, DNMT1 levels are controlled by STAT3. vIL-6 binds gp130, and then activates JAK-STAT3 pathway, which plays a crucial role in KSHV pathogenesis and KS tumor formation [38]. Here, we show that aberrant expression of DNMT1 and disruption of global DNA methylation are the downstream event of JAK-STAT3 activation induced by vIL-6, and it might be one of the mechanisms of KS tumorigenesis. To date, KS is still difficult for treatment. vIL-6 might be a potential target for KS therapy.
